# Enhancement of the Antibiofilm Activity of Nisin against *Listeria monocytogenes* Using Food Plant Extracts

**DOI:** 10.3390/pathogens12030444

**Published:** 2023-03-12

**Authors:** Pitchaya Santativongchai, Phitsanu Tulayakul, Byeonghwa Jeon

**Affiliations:** 1 Division of Environmental Health Sciences, School of Public Health, University of Minnesota, St. Paul, MN 55108, USA; 2 Department of Veterinary Public Health, Faculty of Veterinary Medicine, Kasetsart University, Kamphaeng Saen Campus, Nakhon Pathom 73140, Thailand

**Keywords:** nisin, sage, gallic acid, *Listeria monocytogenes*, antibiofilm

## Abstract

*Listeria monocytogenes* is a foodborne pathogen exhibiting a high mortality rate. In addition to the robust tolerance to environmental stress, the ability of *L. monocytogenes* to develop biofilms increases the risk of contaminating food processing facilities and ultimately foods. This study aims to develop a synergistic approach to better control *Listeria* biofilms using nisin, the only bacteriocin approved as a food preservative, in combination with gallic-acid-rich food plant extracts. Biofilm assays in the presence of nisin and gallic acid or its derivatives revealed that gallic acid significantly decreased the level of biofilm formation in *L. monocytogenes*, whereas ethyl gallate, propyl gallate, and lauryl gallate enhanced biofilm production. As gallic acid is widely distributed in plants, we examined whether extracts from gallic-acid-rich food plants, such as clove, chestnut, oregano, and sage, may generate similar antibiofilm effects. Remarkably, sage extracts enhanced the antibiofilm activity of nisin against *L. monocytogenes*; however, the other tested extracts increased biofilm formation, particularly at high concentrations. Moreover, sage extracts and nisin combinations significantly reduced the biofilm formation of *L. monocytogenes* on stainless steel. Sage is a common food spice and has various beneficial health effects, including antioxidation and anti-cancer properties. The findings in this study demonstrate that sage extracts can be potentially combined with nisin to prevent biofilm production in *L. monocytogenes*.

## 1. Introduction

*Listeria monocytogenes* (*L. monocytogenes*) is a bacterial pathogen implicated in serious foodborne illnesses and outbreaks, particularly in vulnerable populations, such as the elderly, immunocompromised people, and pregnant women [1]. It is a Gram-positive facultative rod bacterium and can grow in a temperature range of −1.5 °C to approximately 50 °C, with an optimum temperature between 30 and 37 °C [2,3]. *L. monocytogenes* infection, called listeriosis, is relatively rare, but potentially fatal, resulting in the development of bacteremia, meningitis, and miscarriage [4], and demonstrates 20–30% mortality rate, which is highest among foodborne pathogens [5,6]. While similar to other bacteria, the growth of *L. monocytogenes* is influenced by environmental conditions such as temperature, pH, and water activity [3], *L. monocytogenes* is ubiquitously prevalent in environments related to food production and processing and is highly tolerant to harsh environmental conditions [7]. Moreover, *L. monocytogenes* tends to produce biofilms and persists in food processing facilities [8]. Biofilms are microbial communities embedded in a self-produced extracellular matrix, which protects the cells and facilitates microbial attachment to food products and packaging materials [9]. Biofilm production not only raises concerns about *Listeria* contamination, but also increases bacterial tolerance to disinfectants and stressors (e.g., heat and desiccation) during food processing, resulting in persistent food contamination [10]. The food industry employs preventive and control strategies, which typically include specific cleaning and accurate sanitation procedures to prevent the adhesion of microorganisms to foods or food-contact surfaces [9,11]. In this regard, complementary alternative strategies, such as natural antimicrobial compounds, enzymes, and bacteriophages, may provide effective measures to control *L. monocytogenes* and its biofilms [9]. Despite the efforts made by the food industry, *L. monocytogenes* is a continuous cause of numerous food recalls and outbreaks [12], indicating that the food industry still demands intervention measures to control *L. monocytogenes* and its biofilms.

Nisin is the only bacteriocin approved as a food preservative [13]. Nisin is highly effective against Gram-positive bacteria, including *Listeria*, *Staphylococcus*, *Clostridium*, and *Bacillus*, by forming pores in the membrane and inhibiting cell wall synthesis through binding to lipid II [14,15]. Because of its potent antimicrobial activity, nisin has been used by the food industry to control *L. monocytogenes* in dairy, meat, and canned products [16,17,18]. In addition, nisin has been reported to inhibit the growth of other species of *Listeria* in combination with some natural organic compounds, including carvacrol, eugenol, cinnamic acid, and thymol [19]. However, the antimicrobial activity of nisin can be limited by multiple factors, including interactions with food components, enzymatic degradation, and pH conditions [13,20]. Studies have been conducted to enhance the antimicrobial activity of nisin by combining it with other natural antimicrobials, such as phages [21,22], lysozymes [23], propolis [17], and citric acid [24]. In our previous studies, we demonstrated that some gallic acid derivatives can potentiate the activity of antibiotics in *Staphylococcus* spp. by increasing drug permeability [25,26,27,28]. Gallic acid is a phenolic acid widely distributed in various kinds of food plants, such as nuts, sage, black tea leaves, walnut, oregano, clove, chicory, and chestnut, and also has various beneficial health functions, including antioxidation, antimicrobial, and anti-obesity properties [29]. Some gallic acid derivatives, such as propyl gallate and octyl gallate, are used as additives in foods, cosmetics, and pharmaceutical products [30,31], and have been suggested for their antimicrobial and antibiofilm effects against *L. monocytogenes* [32,33,34].

Our previous studies raised a research question about whether gallic acid or its derivatives may generate antimicrobial synergy with nisin in *L. monocytogenes*. In this study, we evaluated the antimicrobial synergy between nisin and gallic acid and its derivatives and discovered that gallic acid and sage extracts synergistically increase the antibiofilm activity of nisin against *L. monocytogenes*.

## 2. Materials and Methods

### 2.1. Bacterial Strains

*L. monocytogenes* ATCC 19115 was purchased from the American Type Culture Collection (ATCC, Manassas, VA, USA). *L. monocytogenes* strains SAMN05179388, SAMN03178083, SAMN03198339, and SAMN03198340 were isolated by the Minnesota Department of Health from frozen hash brown (SAMN05179388) and human listeriosis cases (SAMN03178083, SAMN03198339, and SAMN03198340). These strains were cultured aerobically on Brain Heart Infusion (BHI) media (Becton, Dickinson and Company, Sparks, MD, USA) at 37 °C. 

### 2.2. Antimicrobial Susceptibility Test

Microtiter broth dilution assay [35] was used to measure the minimum inhibitory concentration (MIC) of nisin (MilliporeSigma, St. Louis, MO, USA), gallic acid (MilliporeSigma), ethyl gallate (MilliporeSigma), propyl gallate (MilliporeSigma), butyl gallate (Tokyo Chemical Industry Co., Ltd., Tokyo, Japan), octyl gallate (MilliporeSigma), lauryl gallate (MilliporeSigma), and stearyl gallate (Tokyo Chemical Industry Co., Ltd.). The plates were incubated at 37 °C overnight and the results were read.

### 2.3. Biofilm Assays

Oregano (McCormick, Hunt Valley, MD, USA), cloves (Naturevibe Botanicals, Rahway, NJ, USA), chestnut (I LOVE ME attitude, Huntington Beach, CA, USA), and sage (McCormick) were purchased from trusted vendors and were subjected to ethanol extraction, as described previously [36]. Biofilm assays were performed according to the previous study [37], with slight modifications. Briefly, nisin and plant extracts were two-fold serially diluted in a total volume of 50 µL each on the row and column, respectively. Subsequently, 100 µL of each strain of *L. monocytogenes* suspension was added to each well (5 × 10^4^ CFU per well). The plates were incubated overnight at 37 °C. After discarding the bacterial culture from the culture plates, the plates were washed with phosphate-buffered saline (PBS) pH 7.4 twice and stained with 1% crystal violet for 40 min. Subsequently, the crystal violet was removed and the plates were washed thrice with PBS. The stained biofilms were eluted with elution buffer (10% acetic acid and 30% methanol) and measured with a microplate reader (Varioskan™ LUX, ThermoFisher, Waltham, MA, USA) at 595 nm. The biofilm assays were also performed on stainless steel with the combination of nisin and sage against *L. monocytogenes* ATCC 19115 according to a previous report [38]. Briefly, 1 mL of bacterial suspension, which was prepared as described above, was added to a well of a 24-well plate containing stainless steel coupons with a 12.7 mm diameter (Thermo Fisher Scientific, Waltham, MA, USA). After overnight incubation at 37 °C, the stainless coupon was carefully transferred to a fresh 24-well plate with sterilized forceps and was subjected to the biofilm assay as mentioned above. 

### 2.4. Fluorescence Microscopic Analysis of Biofilms

The biofilm formation was also examined with fluorescence microscopy. The biofilms of *L. monocytogenes* ATCC 19115 were aerobically formed on glass slides at 37 °C for 48 h, as described above. The biofilm samples were then washed twice with PBS and fixed with 4% paraformaldehyde (Sigma-Aldrich^®^, St. Louis, MO, USA) at room temperature for 30 min. After that, the samples were washed with PBS and stained with SYTO 9 dye (LIVE/DEAD™ *Bac*Light™ Bacterial Viability Kit, Thermo Fisher Scientific, Waltham, MA, USA). Subsequently, the samples were washed and analyzed with a fluorescence microscope (Olympus BX53, Shinjuku, Tokyo, Japan). The experiments were repeated in triplicate.

### 2.5. Statistical Analysis

Data are reported as the means ± standard deviations. The results were analyzed using one-way ANOVA, followed by Bonferroni’s pos thoc test for multiple comparisons, and Student’s *t*-test for comparisons of the biofilm formation between with and without nisin samples using GraphPad Prism 5 (GraphPad Software, Boston, MA, USA).

## 3. Results

### 3.1. Antibiofilm Synergy between Nisin and Gallic Acid in L. monocytogenes

**Figure 1:**
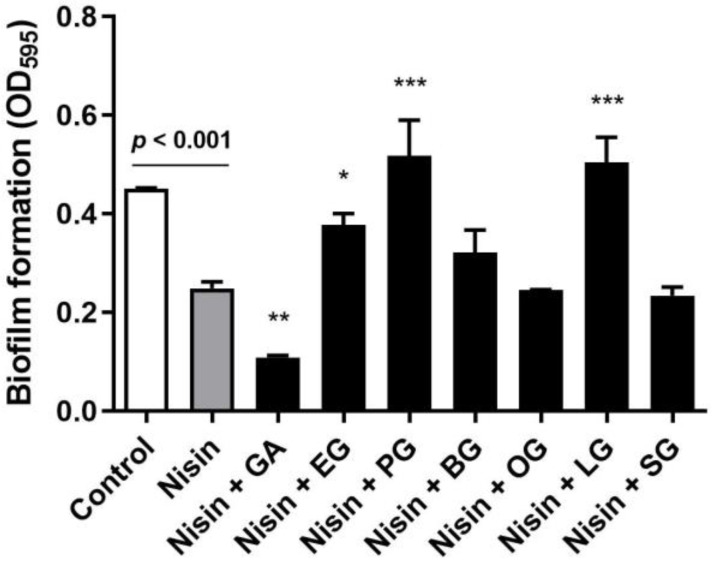
Biofilm formation of *Listeria monocytogenes* ATCC 19115 in the presence of 4 µg/mL of nisin and half MICs of gallic acid or its derivatives, including gallic acid (128 µg/mL), ethyl gallate (64 µg/mL), propyl gallate (32 µg/mL), butyl gallate (32 µg/mL), octyl gallate (16 µg/mL), lauryl gallate (16 µg/mL), and stearyl gallate (256 µg/mL). The data are presented as the means and standard deviations of triplicate samples in a single experiment. The experiment was repeated three times and produced similar results. * *p* < 0.05, ** *p* < 0.01, *** *p* < 0.001 by Student’s *t*-test in comparison with a control.

In order to assess whether gallic acid and its derivatives can generate antibiofilm synergy with nisin, we first measured the level of biofilm production of *L. monocytogenes* in the presence of combinations of nisin and gallic acid or its derivatives, including ethyl gallate, propyl gallate, butyl gallate, octyl gallate, lauryl gallate, and stearyl gallate. Gallic acid and its derivatives showed wide variations in the minimum inhibitory concentrations (MICs) in *L. monocytogenes*: gallic acid (256 µg/mL), ethyl gallate (128 µg/mL), propyl gallate (64 µg/mL), butyl gallate (64 µg/mL), octyl gallate (32 µg/mL), lauryl gallate (32 µg/mL), and stearyl gallate (512 µg/mL). Thus, we measured biofilm formation in *L. monocytogenes* in the presence of nisin and a half MIC of gallic acid or each gallic acid derivative. A sub-MIC concentration was selected for the biofilm assay to avoid bacterial inhibition by high lethal concentrations of the compounds. Gallic acid and its derivatives differentially affected the antibiofilm activity of nisin in *L. monocytogenes*. Notably, gallic acid significantly (*p* < 0.01) reduced the level of biofilm formation compared with the control that was treated with nisin alone (Figure 1). Butyl gallate, octyl gallate, and stearyl gallate did not change the activity, whereas ethyl gallate, propyl gallate, and lauryl gallate rather enhanced biofilm production, indicating that these compounds are antagonistic to the activity of nisin (Figure 1). These results show that gallic acid and its derivatives can influence the antibiofilm activity of nisin differentially, either synergistically, indifferently, or antagonistically, and that gallic acid intensifies the antibiofilm activity of nisin. 

### 3.2. Evaluation of Antibiofilm Synergy between Nisin and Food Plant Extracts Rich in Gallic Acid

**Figure 2:**
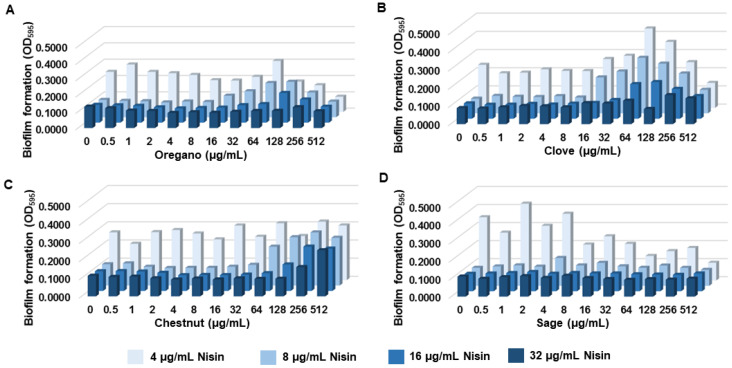
Biofilm formations of *Listeria monocytogenes* ATCC 19115 in the presence of nisin combined with ethanol extracts of oregano (**A**), clove (**B**), chestnut (**C**), and sage (**D**). The data present the means and standard deviations of triplicate samples in a single experiment. The experiment was reported three times and produced similar results.

Gallic acid is a phenolic acid widely distributed in plants and plant-based foods, such as fruits, berries, nuts, and tea [29]. Thus, we hypothesized that extracts from gallic-acid-rich food plants may generate a synergistic antibiofilm activity with nisin, as observed with gallic acid (Figure 1). Using Phenol-Explorer [39], we selected clove, chestnut, oregano, and sage based on their reported high gallic acid contents, which were 458.19 mg/100 g [40], 479.78 mg/100 g [41], 5.15 mg/100 g [42], and 5.25 mg/100 g [42], respectively. In order to evaluate the synergy in antibiofilm activity, we conducted biofilm assays with nisin in combination with extracts from clove, chestnut, oregano, and sage in broad concentration ranges. Interestingly, most of the plant extracts, including oregano, clove, and chestnut, increased the level of biofilm production when used at high concentrations (≥64 µg/mL) (Figure 2A–C). The antagonistic effects of these plant extracts were observed even in the samples treated with high concentrations (e.g., 32 µg/mL) of nisin (Figure 2A–C). In contrast, the combinations of nisin and sage extracts reduced biofilm formation in proportion to increases in the concentration (Figure 2D). 

**Figure 3:**
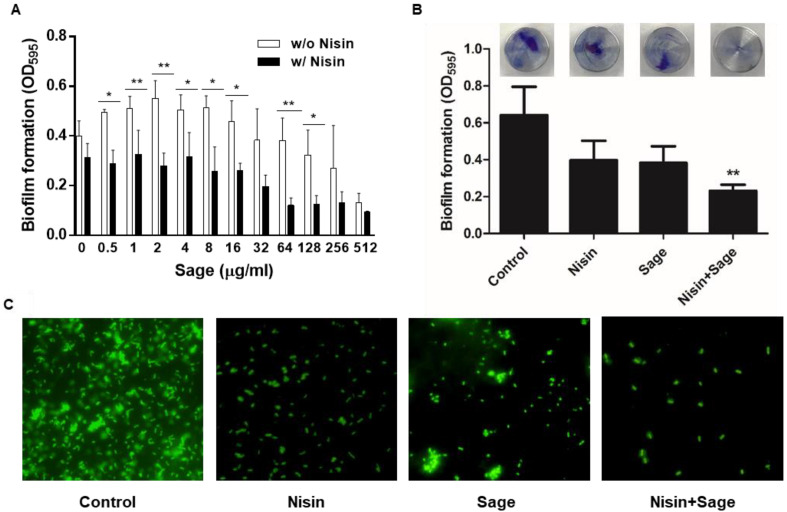
Synergistic anti-biofilm activity of nisin and sage extract combinations. (**A**) Biofilm formations of *Listeria monocytogenes* ATCC 19115 in the presence of 4 µg/mL of nisin with or without sage extract. * *p* < 0.05, ** *p* < 0.01 by Student’s *t*-test. (**B**) Synergistic inhibition of biofilm establishment on stainless steel by the combination of nisin and sage extract. The concentrations of nisin and sage extract used are 4 µg/mL and 128 µg/mL, respectively. ** *p* < 0.01 by one-way ANOVA followed by Bonferroni’s multiple comparison test. (**C**) Synergistic biofilm inhibition by nisin and sage extract observed with fluorescence microscopy after staining with SYTO 9.

The synergy was further investigated with nisin and serially-diluted sage extracts. In the absence of sage extract (control), 4 µg/mL of nisin did not reduce the level of biofilm production; however, biofilm formation was significantly (*p* < 0.05) decreased by nisin in combination with 64 µg/mL of sage extracts (Figure 3A). A synergistic antibiofilm activity between nisin and sage was also observed on stainless steel. A combination of nisin and sage significantly (*p* < 0.01) eliminated biofilm formation on stainless steel compared with the controls (Figure 3B). Consistently, fluorescence microscopic analysis exhibited that the combination of nisin and sage extracts markedly reduced biofilm formation compared with a non-treated control and the samples that were treated with either nisin or sage extracts (Figure 3C). These results demonstrate that sage extracts can increase the antibiofilm activity of nisin in *L. monocytogenes*. Further performing biofilm assays utilizing combinations of 4 µg/mL of nisin and 128 µg/mL of the plant extracts confirmed the results (Supplementary Material Figure S1). The outcomes consistently indicated that the combination of nisin and sage significantly reduced the biofilm formation of *L. monocytogenes*. In contrast, combining nisin with other extracts resulted in increased biofilm levels compared with the control treated with only nisin (Figure S1), suggesting that the presence of these extracts acted antagonistically against the antibiofilm activity of nisin.

### 3.3. Synergistic Antibiofilm Effects of Nisin and Sage Extracts on L. monocytogenes Isolates from Food and Clinical Cases

**Figure 4:**
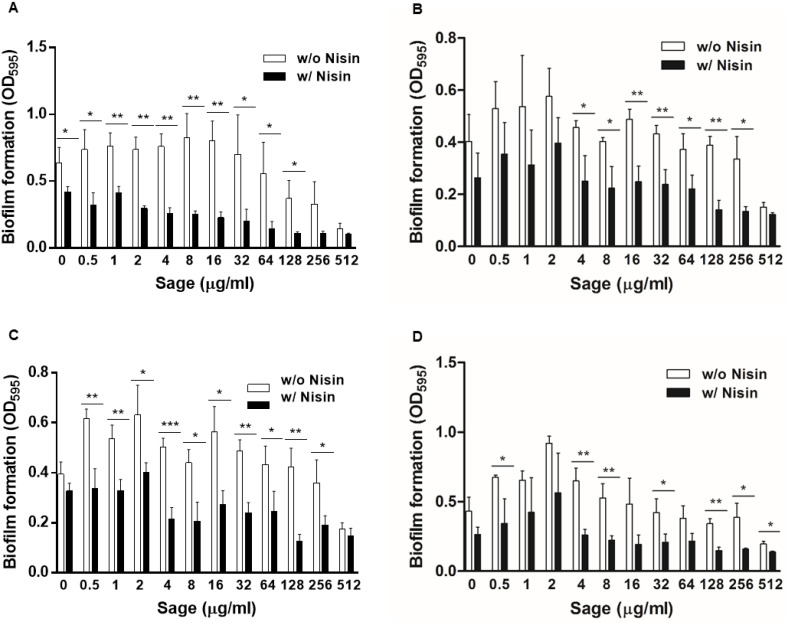
Synergistic anti-biofilm activity of nisin and sage extract combinations in food isolates of *Listeria monocytogenes*, including (**A**) SAMN05179388 (**B**) SAMN03178083, (**C**) SAMN03198339, and (**D**) SAMN03198340. The concentration of nisin used in the experiment was 4 µg/mL. Statistical significance (* *p* < 0.05, ** *p* < 0.01, *** *p* < 0.001) in biofilm levels between with and without nisin was determined by Student’s *t*-test.

We investigated the synergy in antibiofilm activity between nisin and sage extracts with *L. monocytogenes* strains from food and listeriosis cases, which were isolated by the Minnesota Department of Health. Notably, nisin and sage extract combinations showed similar trends of biofilm inhibition in the tested strains. Nisin alone at 4 µg/mL did not inhibit biofilm production, and sage extracts alone reduced biofilm formation only at high concentrations (e.g., 512 µg/mL) (Figure 4). When combined with sage extracts, however, nisin significantly reduced the level of biofilm production (Figure 4). Although there were strain-dependent variations in the levels of biofilm formation, these data confirm that nisin combinations of nisin and sage extracts can inhibit biofilm formation in *L. monocytogenes*.

## 4. Discussion

Food contamination by *L. monocytogenes* is a serious food safety problem impacting both consumers and the food industry [1,5,6]. It is imperative to develop effective intervention measures to address this important public health issue. As nisin is the sole bacteriocin approved as a food preservative [13], various approaches have been made to better control *L. monocytogenes* by further potentiating nisin. Several compounds have been reported to generate antimicrobial synergy in combination with nisin against *L. monocytogenes*, including propolis [17], citric acid [24], sodium diacetate [43], perilla oil [44], and grape seed extract [45]. Based on our previous studies on the antimicrobial synergy of gallic acid derivatives in other Gram-positive pathogens [25,26,27,28], we examined whether gallic acid and its derivatives could enhance the activity of nisin against *L. monocytogenes,* and discovered that gallic acid increases the antibiofilm activity of nisin (Figure 1). 

It has been reported that high concentrations of gallic acid show an antimicrobial activity in *L. monocytogenes* by permeabilizing the bacterial membranes [46] and decreasing extracellular pH [32]. The mode of action of nisin is through pore formation in the membrane and the inhibition of cell wall synthesis [14,15]. In addition, the antimicrobial activity of nisin is strongly affected by pH [47] and is increased az acidic pH [48]. Presumably, the synergy between gallic acid and nisin may be associated with membrane permeability and pH. In a previous study, it was discovered that higher concentrations of nisin and gallic acid, alone or in combination, effectively decreased the growth of *L. monocytogenes* and lipid peroxidation [49]. Butyl gallate, a derivative of gallic acid, exhibited synergistic effects with nisin compared to other derivatives [50]. Additionally, the combination of gallic acid and certain natural extracts showed antimicrobial synergy against other pathogenic bacteria, including *Pseudomonas aeruginosa* and *Staphylococcus aureus* [51,52].

The findings in this study demonstrated that sage extracts increased the antibiofilm activity of nisin in *L. monocytogenes*. Sage (*Salvia officinalis*) is widely used as a spice due to its seasoning properties and has been traditionally used for treating various kinds of disorders, including dizziness, dyspepsia, ulcers, and gout [53]. In addition, sage has beneficial health effects, such as anticancer [54] and antidementia properties [55]. Studies have shown that sage has antimicrobial effects on *L. monocytogenes* [56,57]. Moreover, sage essential oil has antimicrobial properties against a variety of pathogens, including *Salmonella* and *Serratia* [58], and exhibits antibiofilm activity against *P. aeruginosa* [59]. 

Studies have shown that other natural extracts containing gallic acid, including pomegranate tree [60], oregano oil [61], and clove oil [62], have an anti-listerial activity. We also evaluated the antibiofilm activity of nisin in combination with extracts of clove, chestnut, and oregano, which are known to have high gallic acid levels [40,41,42]. Our findings suggest that those extracts may interfere with the antibiofilm activity of nisin against *L. monocytogenes* and that these combinations were rather antagonistic (Figure S1). These previous studies employed oregano and clove oils [61,62], whereas in our study, ethanol extracts were utilized, which could have varying components due to the different solubility properties. However, only sage extracts synergistically decreased biofilm formation in *L. monocytogenes*, and the other tested extracts generated antagonism to the activity of nisin, particularly when used at high concentrations (Figure 2). These findings suggest that the activity of nisin can be significantly influenced by food components and that the use of nisin and sage could be a new combination to control the growth and biofilm formation of *L. monocytogenes* in foods. 

Strain variations can impact the antimicrobial susceptibility of *L. monocytogenes* [63]. In our study, we evaluated the efficacy of combinations of nisin and sage against several *L. monocytogenes* isolates obtained from clinical cases and food products. The combination of nisin and sage displayed effectiveness against different strains of *L. monocytogenes* (Figure 4), indicating that it could be a viable strategy for controlling the growth and biofilm formation of a diverse range of *L. monocytogenes* strains in the food industry. However, further validation through scaled-up experiments using a larger number of strains isolated from various sources, including food products and processing environments, is necessary.

In conclusion, the combination of gallic acid and sage extract was found to significantly enhance the antibiofilm activity of nisin against *L. monocytogenes*. As gallic acid, sage, and nisin are generally recognized as safe (GRAS) compounds [16,29,53], these combinations could potentially serve as additives in food products and/or antifouling agents during food processing to prevent the biofilm formation of *L. monocytogenes*. However, given the observed antagonistic activities of nisin in conjunction with oregano, cloves, and chestnut, their use in food processing should be cautiously monitored to control *Listeria*. Further research is needed to investigate the molecular mechanisms underlying their synergistic effects.

## Data Availability

Not applicable.

## Supplementary Materials

The following supporting information can be downloaded at: https://www.mdpi.com/article/10.3390/pathogens12030444/s1, Figure S1: Biofilm formation of *Listeria monocytogenes* ATCC 19115 in the presence of 4 µg/mL of nisin combined with 128 µg/mL of ethanol extracts of oregano, cloves, chestnut, and sage.
