# Supplementary material for: Enhancement of the Antibiofilm Activity of Nisin against Listeria monocytogenes Using Food Plant Extracts
Source: Pathogens. 2023 Mar 12;12(3):444. doi: 10.3390/pathogens12030444 (PMC10056046; doi:10.3390/pathogens12030444)
Supplement: Supplementary file 1 [file pathogens-12-00444-s001.zip › pathogens-2204082-supplementary.pdf]

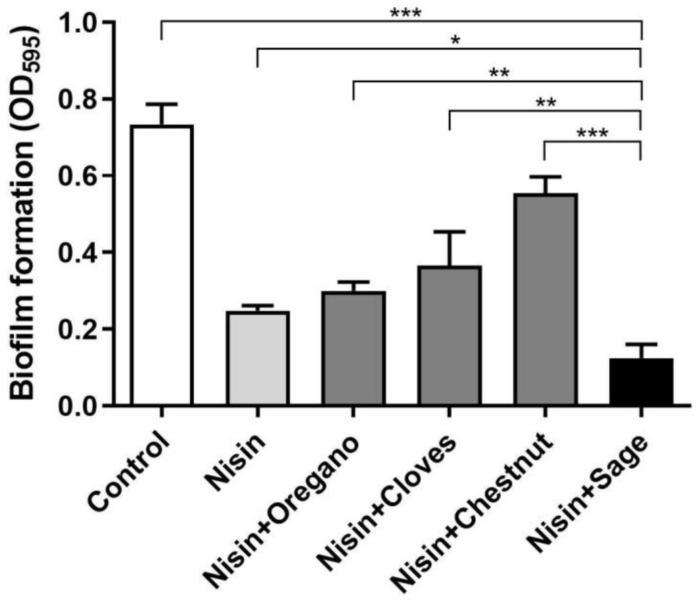

Figure S1. Biofilm formation of *Listeria monocytogenes* ATCC 19115 in the presence of 4 μg/mL of nisin combined with 128 μg/mL of ethanol extracts of oregano, cloves, chestnut, and sage. The data are presented as the means and standard deviations of triplicate samples in a single experiment. *: $p < 0.05$, **: $p < 0.01$, ***: $p < 0.001$.
